# Correction to: Clinical, radiological and functional outcomes in patients with SARS-CoV-2 pneumonia: a prospective observational study

**DOI:** 10.1186/s12890-021-01686-1

**Published:** 2021-11-01

**Authors:** Pietro Gianella, Elia Rigamonti, Marco Marando, Adriana Tamburello, Lorenzo Grazioli Gauthier, Gianluca Argentieri, Carla Puligheddu, Alberto Pagnamenta, Marco Pons, Tanja Fusi-Schmidhauser

**Affiliations:** 1 Department of Internal Medicine, Ospedale Regionale di Lugano, Ente Ospedaliero Cantonale, Via Tesserete 46, 6900 Lugano, Switzerland; 2 Division of Pneumology, Ospedale Regionale di Lugano, Ente Ospedaliero Cantonale, Lugano, Switzerland; 3 IIMSI - Radiology Department, Ospedale Regionale di Lugano, Ente Ospedaliero Cantonale, Lugano, Switzerland; 4 Department of Intensive Care, Intensive Care Unit Ospedale Regionale di Mendrisio, Ente Ospedaliero Cantonale, Lugano, Switzerland; 5 Unit of Biostatistics, Ente Ospedaliero Cantonale, BellinzonaLugano, Switzerland; 6 Division of Pneumology, University of Geneva, Geneva, Switzerland

## Correction to: BMC Pulm Med (2021) 21:136 10.1186/s12890-021-01509-3

Following publication of the original article, the authors flagged that a calculation error had been made in the calculation of quality of life scores in Table 5 (specifically, of SF-12).

**Table 5:** QoL assessment at three months

	Normal value	Overall (39)	CT improving at 3 mo (31)	CT not improving at 3 mo (8)	*P* value
St George symptoms (median and 25th–75th)	12 (9–15)	16.3 (10.4–29.8)	16.3 (10.4–29.8)	20.5 (6.5–31.7)	0.84
St George activity (median and 25th–75th)	9 (7–12)	19 (12.2–41.4)	18.1 (6.3–35.5)	40.1 (18.8–69.6)	0.08
St George impact (median and 25th–75th)	2 (1–3)	4 (0–11.5)	2 (0–8.2)	12.2 (0–414)	0.13
St George total (median and 25th–75th)	6 (5–7)	9.9 (7.7–21)	9.9 (7.2–16.2)	20.4 (8.1–50.5)	0.16
Abnormal St. George total (n and %)	–	31 (79.5)	24 (77.4)	7 (87.5)	1
SF-12 physical (median and 25th–75th)	> 50	50.5 (36.1–55)	53.1 (41.9–56)	32.5 (28.9–52.5)	0.051
SF-12 mental (median and 25th–75th)	> 50	54.9 (43.5–59.9)	57.5 (49–59.9)	53.1 (41.4–58.9)	0.64
Abnormal SF-12 physical (n and %)	–	19 (48.7)	14 (45.2)	5 (62.5)	0.45
Abnormal SF-12 mental (n and %)	–	12 (30.8)	9 (29)	3 (37.5)	0.68

The table has been corrected in the published article and it may be found below.

As before, 31 patients (79.5%) presented an abnormal total score in the St. George’s Respiratory Questionnaire. Concerning SF-12, the authors have split it in its physical and mental components and corrected a critical error in calculating the scores. At 3 months, 19 (48.7%) and 12 (30.8%) patients had an abnormal SF-12 score in physical and mental components, respectively. Still, the authors have not found any significant difference in SF-12 scores among those who showed radiological improvement at 3 months. The other findings related to quality-of-life outcomes remained similarly unchanged. Finally, the authors have updated the normal values choosing those widely accepted in literature, removed the speculative analysis comparing overall results with the aforementioned normal values and, since the distribution was not normal, chosen to describe the data as median and interquartile range.

The authors thank you for reading this correction and apologize for any inconvenience caused.

